# Population‐based assessment of risks for severe COVID‐19 disease outcomes

**DOI:** 10.1111/irv.12901

**Published:** 2021-08-25

**Authors:** Ousseny Zerbo, Ned Lewis, Bruce Fireman, Kristin Goddard, Jacek Skarbinski, James J. Sejvar, Eduardo Azziz‐Baumgartner, Nicola P. Klein

**Affiliations:** ^1^ Vaccine Study Center Kaiser Permanente Northern California Oakland California USA; ^2^ Department of Infectious Diseases Kaiser Permanente Northern California Oakland California USA; ^3^ COVID‐19 Response Team U.S. Centers for Disease Control and Prevention Atlanta Georgia USA

**Keywords:** comorbidities, COVID‐19, race/ethnicity, risk factors, severe disease

## Abstract

Among approximately 4.6 million members of Kaiser Permanente Northern California, we examined associations of severe COVID‐19 with demographic factors and comorbidities. As of July 23, 2021, 16 182 had been hospitalized, 2416 admitted to an ICU, and 1525 died due to COVID‐19. Age was strongly associated with hospitalization, ICU admission, and death. Black persons and Hispanic ethnicity had higher risk of death compared with Whites. Among the comorbidities examined, Alzheimer's disease was associated with the highest risk for hospitalization (aHR 3.19, CI: 2.88–3.52) and death (aHR 4.04, CI: 3.32–4.91). Parkinson's disease had the second highest risk of death (aHR = 2.07, CI: 1.50–2.87).

## 1 INTRODUCTION

Information on risk of severe coronavirus disease 2019 (COVID‐19) is important to identify whom might benefit most from COVID‐19 prevention including vaccination and assertive treatment. Previous studies reported that older age^1^, ^2^ and comorbidities including asthma, chronic obstructive pulmonary disease (COPD), diabetes, and heart and renal disease are associated with severe COVID‐19.^3^, ^4^, ^5^, ^6^, ^7^ However, association between some neurological condition such as Parkinson's disease, demyelinating disorders, and epilepsy have not been examined extensively. Furthermore, non‐White racial and ethnic persons are reportedly disproportionally affected by COVID‐19.^6^, ^8^, ^9^, ^10^, ^11^, ^12^ Previous studies on risk factors were conducted when vaccines were not widely available. This study expands on knowledge from earlier studies by using population‐based information from Kaiser Permanente in Northern California (KPNC) where comprehensive databases permit assessment of risks of testing positive for SARS‐CoV‐2, COVID‐19 hospitalization, admission to an intensive care unit (ICU), and death in relation to demographic factors and comorbidities and assess whether risk factors changed in the presence of COVID‐19 vaccines.

## 2 MATERIALS AND METHODS

### 2.1 Setting and population

This study was conducted at KPNC, an integrated healthcare system with a stable population of approximately 4.6 million members who receive nearly all their healthcare at KPNC facilities. Clinical databases include comprehensive information updated daily on diagnoses in the outpatient, emergency department, and inpatient settings and on prescriptions and laboratory tests.

### 2.2 Study design

Using KPNC clinical databases, we conducted near‐real‐time surveillance of the pandemic, monitoring testing, hospitalization, ICU admission, and death. We report here on association between severe COVID‐19 and risk factors included in our weekly surveillance from January 1, 2020, through July 23, 2021.

### 2.3 Demographic factors and comorbidities

Demographic factors examined in relation to COVID‐19 include age, sex, self‐reported race/ethnicity, and type of insurance (subsidized [i.e., government subsidized other than Medicare] or non‐subsidized). The comorbidities examined include body mass index (BMI), diabetes, essential hypertension, renal disease, asthma, ischemic heart disease, COPD, pneumonia, cerebral infarction, Alzheimer's disease, Parkinson's disease, extrapyramidal and movement disorders, demyelinating disorders, and epilepsy. Comorbidities were included only if diagnosed at least once before the pandemic, from October 2015 through January 2020. Comorbidities were classified by International Classification of Diseases, Tenth Revision codes. Many but not all of these comorbidities were associated with COVID‐19 in earlier reports.

### 2.4 COVID‐19‐related outcomes

We examined three indicators of severity (hospitalization, ICU admission, and mortality) stratified by the presence or absence of severe acute respiratory syndrome coronavirus 2 (SARS‐CoV‐2) infection confirmed by polymerase chain reaction (PCR).

### 2.5 Statistical analysis

We described testing positive, COVID‐19 hospitalizations, ICU admissions, and mortality by sex, age‐group, race/ethnicity, and comorbidities. We used Cox regression to assess associations between testing positive, severe COVID‐19 outcomes with demographic factors and comorbidities. To further assess association of severe COVID with demographic and comorbidities, we also conducted additional analyses restricted to people who tested positive and to those who were hospitalized only. We used Cox models conditioned on a calendar timeline and 14 geographic service areas.

To assess whether risk factors associated with severe COVID‐19 evolved during the course of the pandemic, we conducted supplemental analyses by dividing the study into three time periods: The first period included data from January 1, 2020, through October 30, 2020, when no vaccines were available; the second was from November 1, 2020, through February 28, 2021, when limited vaccine doses were available and during the biggest surge of SARS‐CoV‐2 infection; and the third was from March 1, 2021, through July 23, 2021, when vaccines were widely available. After COVID‐19 vaccines became available, these supplemental analyses censored people when they received the vaccine.

KPNC Institutional Review Board approved and determined this project to be exempt from human subject review under the Federal Regulations.

## 3 RESULTS

The study included approximately 4.6 million KPNC members, all ages. As of July 23, 2021, 219 001 tested positive for SARS‐CoV‐2, 16 182 had been hospitalized due to COVID‐19, 2416 admitted to an ICU, and 1525 died. The incidence of testing positive for SARS‐CoV‐2, hospitalization, admission to an ICU, and death varied during the study period, with the highest rate of each occurring during December 2020 to January 2021 (~400/100000 person‐weeks for testing positive, 22/100000 person‐weeks for hospitalization, 3.5/100000 person‐weeks for ICU admission, and 3.5/100000 person‐weeks for death; Figure S1). The incidence of testing positive for SARS‐CoV‐2 was highest among people ages 20–29 years (89.7/100000 person‐weeks) and among Hispanics (121.8/100000 person‐weeks). Age and race/ethnicity were strongly associated with severe COVID‐19. Hospitalizations per 100 000 person‐weeks increased markedly with age from 0.3 at ages 0–9 to 25.7 at ages ≥90 (Table 1).

**TABLE 1:** Demographics characteristics and comorbidities of the surveillance population and people with severe COVID‐19, Kaiser Permanente Northern California, January 1, 2020, to July 23, 2021

	*KPNC population N* = 4 579 858	*Positive test N* = 219 001		Severe COVID‐19‐related events					
				*Hospitalization N* = 16 182		*Admitted to ICU N* = 2416		*Deaths N* = 1525	
	*N* (%)	*N* (%)	Incidence^a^	*N* (%)	Incidence^a^	*N* (%)	Incidence^a^	*N* (%)	Incidence^a^
Sex									
Female	2 451 554 (51.1)	115 321 (52.7)	66.4	7902 (48.8)	4.6	943 (39.0)	0.5	646 (42.4)	0.4
Male	2 347 305 (48.9)	103 680 (47.3)	63.4	8280 (51.2)	5.1	1473 (61.0)	0.9	879 (57.6)	0.5
Age category									
0–9	552 388 (11.5)	16 513 (7.5)	44.4	123 (0.8)	0.3	33 (1.4)	0.1	0 (0.0)	0.0
10–19	537 746 (11.2)	25 016 (11.4)	63.8	200 (1.2)	0.5	36 (1.5)	0.1	1 (0.1)	0.0
20–29	687 603 (14.3)	38 676 (17.7)	89.7	962 (5.8)	2.2	72 (3.0)	0.2	4 (0.3)	0.0
30–39	754 936 (15.7)	40 623 (18.5)	79.5	1582 (9.8)	3.1	153 (6.3)	0.3	17 (1.1)	0.0
40–49	647 234 (13.5)	36 034 (16.5)	78.0	2190 (13.5)	4.7	305 (12.6)	0.7	65 (4.3)	0.1
50–59	639 920 (13.3)	31 779 (14.5)	68.4	3219 (19.9)	6.9	545 (22.6)	1.2	207 (13.6)	0.5
60–69	531 071 (11.1)	18 611 (8.5)	47.0	3206 (19.8)	8.1	596 (24.7)	1.5	328 (21.5)	0.8
70–79	301 973 (6.3)	7845 (3.6)	33.3	2552 (15.8)	10.8	442 (18.3)	1.9	386 (25.3)	1.6
80–89	119 233 (2.5)	3186 (1.5)	35.2	1671 (10.3)	18.5	195 (8.1)	2.2	339 (22.2)	3.8
90+	26 755 (0.6)	718 (0.3)	38.7	477 (2.9)	25.7	39 (1.6)	2.1	178 (11.7)	9.6
Race/ethnicity									
White	1 891 899 (39.4)	62 532 (28.6)	45.8	5072 (31.3)	3.7	599 (24.8)	0.4	571 (37.4)	0.4
Black	312 614 (6.5)	15 758 (7.2)	71.5	1654 (10.2)	7.5	252 (10.4)	1.1	153 (10.0)	0.7
Asian	929 812 (19.4)	30 277 (13.8)	44.8	2638 (16.3)	3.9	458 (19.0)	0.7	223 (14.6)	0.3
Hawaiian/Pacific Islander	38 073 (0.8)	2392 (1.1)	89.7	253 (1.6)	9.5	42 (1.7)	1.6	12 (0.8)	0.5
Native American or Alaska Native	20 424 (0.4)	932 (0.4)	65.6	71 (0.4)	5.0	4 (0.2)	0.3	7 (0.5)	0.5
Multiracial	71 559 (1.5)	2693 (1.2)	52.2	202 (1.2)	3.9	28 (1.2)	0.5	30 (2.0)	0.6
Hispanic	1 070 481 (22.3)	90 628 (41.4)	121.8	6065 (37.5)	8.2	998 (41.3)	1.3	521 (34.2)	0.7
Unknown	463 997 (9.7)	13 789 (6.3)	50.4	227 (1.4)	0.8	35 (1.4)	0.1	8 (0.5)	0.0
Comorbidities^b^									
Body mass index^c^									
Underweight	84 127 (1.8)	2451 (1.1)	38.9	172 (1.1)	2.7	17 (0.7)	0.3	27 (1.8)	0.4
Normal	1 162 851 (24.2)	40 833 (18.6)	47.0	1934 (12.0)	2.2	210 (8.7)	0.2	268 (17.6)	0.3
Overweight	992 168 (20.7)	48 814 (22.3)	65.2	3801 (23.5)	5.1	531 (22.0)	0.7	394 (25.8)	0.5
Obese	1 039 639 (21.7)	71 665 (32.7)	92.5	7851 (48.5)	10.1	1304 (54.0)	1.7	704 (46.2)	0.9
Unknown	1 520 074 (31.7)	55 238 (25.2)	59.9	2424 (15.0)	2.6	354 (14.7)	0.4	132 (8.7)	0.1
Diabetes	389 570 (8.1)	23 078 (10.5)	78.5	5591 (34.6)	19.0	1042 (43.1)	3.5	719 (47.1)	2.5
Essential hypertension	855 269 (17.8)	39 350 (18.0)	60.5	8321 (51.4)	12.8	1419 (58.7)	2.2	1155 (75.7)	1.8
Renal disease	208 204 (4.3)	8622 (3.9)	54.9	3445 (21.3)	21.9	603 (25.0)	3.8	611 (40.1)	3.9
Asthma	563 559 (11.7)	31 344 (14.3)	74.1	2986 (18.5)	7.1	464 (19.2)	1.1	287 (18.8)	0.7
Ischemic heart disease	122 644 (2.6)	5010 (2.3)	54.3	1821 (11.3)	19.7	296 (12.3)	3.2	335 (22.0)	3.6
COPD	67 042 (1.4)	2575 (1.2)	51.4	1050 (6.5)	21.0	149 (6.2)	3.0	170 (11.1)	3.4
Pneumonia (history)	185 941 (3.9)	9432 (4.3)	67.7	1830 (11.3)	13.1	315 (13.0)	2.3	302 (19.8)	2.2
Cerebral infarction	25 107 (0.5)	1125 (0.5)	61.1	498 (3.1)	27.1	91 (3.8)	4.9	95 (6.2)	5.2
Alzheimer's disease	12 308 (0.3)	616 (0.3)	73.9	449 (2.8)	53.9	29 (1.2)	3.5	130 (8.5)	15.6
Parkinson's disease	9100 (0.2)	325 (0.1)	48.4	172 (1.1)	25.6	22 (0.9)	3.3	39 (2.6)	5.8
Extrapyramidal and movement disorders	43 791 (0.9)	1957 (0.9)	59.2	480 (3.0)	14.5	67 (2.8)	2.0	72 (4.7)	2.2
Demyelinating disorders	7623 (0.2)	328 (0.1)	56.4	67 (0.4)	11.5	9 (0.4)	1.6	7 (0.5)	1.2
Epilepsy	33 275 (0.7)	1630 (0.7)	66.5	340 (2.1)	13.9	45 (2.2)	2.2	48 (3.1)	2.0
Type of insurance									
Subsidized insurance^d^	468 159 (9.8)	29204 (13.1)	87.3	1779 (11.0)	5.3	294 (12.2)	0.9	120 (7.9)	0.4

Abbreviations: BMI, body mass index; COPD, chronic obstructive pulmonary disease; ICU, intensive care unit.

^a^ Incidence rates/100000 person‐weeks.

^b^ Based on ICD‐10 codes recorded in medical record.

^c^ BMI categories: underweight = BMI < 18.5; normal weight = BMI between 18.5 and24.9; overweight = BMI between 25 and 29.9; obesity = BMI ≥ 30.

^d^ Includes those who cannot afford the regular insurance rates and are not covered by commercial insurance or Medicare.

Black persons represented 6.5% of KPNC members but 10.2% of COVID‐19 hospitalizations, 10.4% of ICU admissions, and 10% of deaths. Hispanic persons represented 22.3% of the KPNC members, but 37.5% of hospitalizations, 41.3% of ICU admissions, and 34.2% of deaths (Table 1).

The adjusted hazard ratios (aHR) for severe COVID‐19 increased markedly with increasing age (Table 2). Compared with ages 40–49 years, risk of death due to COVID‐19 was more than twice as high at ages 50–59 (aHR = 2.76, 95% confidence interval [CI]: 2.09–3.65) and more than 32 times higher at ages ≥90 (aHR = 32.54, CI: 23.68–44.71).

**TABLE 2:** Factors associated with testing positive for SARS‐CoV‐2, COVID‐19 hospitalization, admission to an intensive care unit, and death, Kaiser Permanente Northern California, January 1, 2020, to July 23, 2021 (*N* ~ 4.6 million)

	Positive test	Hospitalization	Admitted to ICU	Deaths
	Hazard ratio (95% CI)	Hazard ratio (95% CI)	Hazard ratio (95% CI)	Hazard ratio (95% CI)
Sex				
Female	Reference	Reference	Reference	Reference
Male	**0.97 (0.96–0.98)**	**1.23 (1.19–1.27)**	**1.77 (1.63–1.92)**	**1.75 (1.57–1.94)**
Age category in year				
0–9	**0.59 (0.58–0.60)**	**0.07 (0.06–0.09)**	**0.17 (0.12–0.25)**	‐
10–19	**0.81 (0.80–0.82)**	**0.12 (0.10–0.14)**	**0.16 (0.11–0.23)**	**0.02 (0.00–0.14)**
20–29	**1.18 (1.17–1.20)**	**0.54 (0.50–0.58)**	**0.31 (0.24–0.41)**	**0.08 (0.03–0.21)**
30–39	**1.08 (1.07–1.10)**	**0.75 (0.70–0.80)**	**0.55 (0.45–0.67)**	**0.28 (0.16–0.47)**
40–49	reference	Reference	Reference	Reference
50–59	**0.86 (0.85–0.88)**	**1.32 (1.25–1.40)**	**1.56 (1.35–1.80)**	**2.76 (2.09–3.65)**
60–64	**0.67 (0.66–0.69)**	**1.44 (1.35–1.53)**	**1.78 (1.51–2.09)**	**4.05 (3.02–5.44)**
65–69	**0.50 (0.49–0.51)**	**1.37 (1.28–1.47)**	**1.87 (1.57–2.21)**	**4.98 (3.71–6.68)**
70–74	**0.42 (0.41–0.43)**	**1.50 (1.39–1.61)**	**1.83 (1.53–2.20)**	**6.11 (4.55–8.21)**
75–79	**0.39 (0.38–0.48)**	**1.79 (1.65–1.93)**	**2.21 (1.82–2.68)**	**8.80 (6.53–11.85)**
80–84	**0.40 (0.38–0.42)**	**2.04 (1.87–2.22)**	**2.18 (1.75–2.73)**	**10.25 (7.53–13.96)**
85–89	**0.43 (0.41–0.46)**	**2.77 (2.52–3.04)**	**1.75 (1.30–2.35)**	**16.83 (12.30–23.01)**
90+	**0.48 (0.44–0.52)**	**3.40 (3.05–3.80)**	**2.33 (1.63–3.32)**	**32.54 (23.68–44.71)**
Race/ethnicity				
White	Reference	Reference	Reference	Reference
Black	**1.37 (1.35–1.40)**	**1.89 (1.78–2.00)**	**2.24 (1.92–2.61)**	**1.75 (1.45–2.11)**
Asian	1.02(1.00–1.03)	**1.54 (1.47–1.62)**	**2.35 (2.06–2.67)**	**1.39 (1.18–1.65)**
Hawaiian/Pacific Islander	**1.76 (1.69–1.83)**	**2.83 (2.49–3.21)**	**3.75 (2.73–5.14)**	1.44 (0.81–2.56)
Native American or Alaska Native	**1.27 (1.19–1.35)**	**1.39 (1.10–1.76)**	0.65 (0.24–1.73)	1.57 (0.75–3.31)
Multiracial	**1.13 (1.09–1.18)**	**1.32 (1.14–1.52)**	**1.57 (1.07–2.29)**	**1.62 (1.12–2.35)**
Hispanic	**2.36 (2.33–2.38)**	**2.70 (2.60–2.81)**	**3.80 (3.42–4.23)**	**2.62 (2.31–2.97)**
Unknown	**1.11 (1.09–1.13)**	**0.46 (0.40–0.53)**	**0.66 (0.47–0.94)**	**0.34 (0.17–0.68)**
Comorbidities^a^				
Body mass index^b^				
Underweight	**0.87 (0.84–0.91)**	**1.45 (1.24–1.70)**	1.47 (0.89–2.41)	**1.37 (0.92–2.04)**
Normal	Reference	Reference	Reference	Reference
Overweight	**1.27 (1.25–1.28)**	**1.31 (1.24–1.39)**	**1.55 (1.32–1.82)**	1.01 (0.86–1.18)
Obese	**1.50 (1.48–1.52)**	**2.27 (2.15–2.40)**	**3.11 (2.66–3.63)**	**1.82 (1.56–2.12)**
Unknown	1.00 (0.98–1.01)	**1.27 (1.19–1.35)**	**1.92 (1.60–2.31)**	1.26 (1.00–1.58)
Diabetes	**1.26 (1.24–1.28)**	**1.76 (1.69–1.83)**	**2.01 (1.82–2.21)**	**1.66 (1.48–1.86)**
Essential hypertension	1.02 (1.0–1.04)	**1.23 (1.18–1.28)**	**1.41 (1.27–1.57)**	**1.51 (1.31–1.74)**
Renal disease	**1.05 (1.02–1.07)**	**1.44 (1.38–1.51)**	**1.64 (1.47–1.84)**	**1.60 (1.42–1.81)**
Asthma	**1.05 (1.04–1.07)**	**1.14 (1.09–1.18)**	**1.16 (1.04–1.29)**	1.08 (0.94–1.23)
Ischemic heart disease	**1.11 (1.08–1.14)**	**1.17 (1.10–1.23)**	1.09 (0.95–1.25)	**1.20 (1.05–1.37)**
COPD	**1.08 (1.03–1.12)**	**1.37 (1.28–1.46)**	**1.22 (1.02–1.45)**	**1.21 (1.02–1.43)**
Pneumonia (history)	**1.19 (1.16–1.22)**	**1.68 (1.60–1.77)**	**1.90 (1.67–2.16)**	**1.88 (1.63–2.16)**
Cerebral infarction	**1.13 (1.07–1.20)**	**1.48 (1.35–1.63)**	**1.68 (1.35–2.08)**	**1.59 (1.29–1.97)**
Alzheimer's disease	**2.11 (1.94–2.29)**	**3.19 (2.88–3.52)**	**1.65 (1.13–2.42)**	**4.04 (3.32–4.91)**
Parkinson's disease	**1.24 (1.11–1.39**)	**1.80 (1.55–2.10)**	**1.55 (1.01–2.38)**	**2.07 (1.50–2.87)**
Extrapyramidal and movement disorders	1.02 (0.97–1.06)	**1.25 (1.14–1.38)**	1.20 (0.94–1.54)	1.25 (0.98–1.59)
Demyelinating disorders	0.90 (0.80–1.00)	**1.75 (1.37–2.22)**	**1.69 (0.88–3.26)**	2.03 (0.97–4.28)
Epilepsy	0.95 (0.91–1.00)	**1.69 (1.51–1.88)**	**1.77 (1.35–2.33)**	**1.81 (1.35–2.42)**
Type of insurance				
Subsidized insurance^c^	**1.14 (1.13–1.16)**	**1.52 (1.45–1.60)**	**1.76 (1.55–2.00)**	**1.52 (1.25–1.84)**

*Note*: Bold indicates that findings are statistically significant and that 95% CI does not include the null value.

Abbreviations: BMI, body mass index; COPD, chronic obstructive pulmonary disease; ICU = intensive care unit.

^a^ Based on ICD‐10 codes recorded in medical record

^b^ BMI categories: underweight = BMI < 18.5; normal weight = BMI between 18.5 and 24.9; overweight = BMI between 25 and 29.9; obesity = BMI ≥ 30.

^c^ Includes those who cannot afford the regular insurance rates and are not covered by commercial insurance or Medicare.

Non‐White racial and ethnic groups had markedly increased risk of severe COVID‐19 compared with White person. Adjusted for comorbidities and other demographic factors, HR estimates were 1.89, 2.24, and 1.75 for the association of Black race with hospitalization, ICU, and death, respectively. The HR estimates were 2.70, 3.80, and 2.62 for the association of Hispanic ethnicity with hospitalization, ICU, and death, respectively (Table 2).

Alzheimer's disease was the comorbidity most strongly associated with testing positive for SARS‐CoV‐2 (aHR = 2.11, CI: 1.94–2.29), hospitalization (aHR = 3.19, CI: 2.88–3.52), and death (aHR = 4.04, CI: 3.32–4.91) (Table 2). Obesity was strongly associated with hospitalization (aHR = 2.27, CI:2.15–2.40), ICU admission (aHR = 3.11, CI: 2.66–3.53), and death (aHR = 1.82, CI: 1.56–2.12) as well as demyelinating disorders (hospitalization aHR = 1.75, CI: 1.37–2.22; death aHR = 2.03, CI: 0.97–4.38). COPD was moderately associated with hospitalization (aHR = 1.37, CI: 1.28–1.46) and death (aHR = 1.21, CI: 1.02–1.43). Asthma (aHR1.14, CI: 1.09–1.18), essential hypertension (aHR = 1.23, CI 1.18–1.28), and ischemic heart disease (aHR 1.17, CI: 1.10–1.23) modestly elevated the hazard of hospitalization (Table 2).

Supplemental analyses by time period based on vaccine availability were not substantially different. Black persons and Hispanic ethnicity remained consistently associated with hospitalization, ICU admission, and death before and after vaccines were widely available. Before vaccines were widely available, aHR were 2.13, 2.70, and 2.43 higher for hospitalization, ICU admission, and death, respectively, among Black persons compared with White persons. After wide availability of vaccines, aHR were 2.12, 2.33 and 1.73 respectively (Table S1).

Among people who tested positive for SARS‐CoV‐2, Blacks, Asian, multiracial, and Hispanic persons were at increased risk of hospitalization, admission to an ICU, and death compared with White persons after adjustment for age and comorbidities (Table S2). Among hospitalized people who tested positive for SARS‐CoV‐2, Hispanic persons were at increased risk of admission to an ICU (aHR = 1.47, CI 1.30–1.67) and death (aHR = 1.37, CI 1.18–1.58) compared with White persons (Table S3).

## 4 DISCUSSION

In this large population‐based study, increasing age was strongly associated with risk of hospitalization, ICU admission, and death. Non‐White racial and ethnic groups were at markedly higher risk of severe COVID‐19. The risk of severe COVID was strongly associated with some comorbidities but not with others. Even after wide availability of COVID‐19 vaccines, the pattern of associations of severe COVID‐19 with the risk factors was stable over time.

Although our results are consistent with previous studies reporting associations of age and race/ethnicity with severe COVID‐19,^12^, ^13^, ^14^ our results pertain to three levels of severity—hospitalization, ICU use, and death—and were adjusted for age, race/ethnicity, sex, comorbidities, and service area. Unlike a meta‐analysis suggesting that the association of age with severe COVID‐19 is much weaker when adjusted for age‐related comorbidities,^10^ our findings suggest age and race/ethnicity were associated with disease severity, even after adjustment for each other and for comorbidities, socioeconomic status, and geographic service area. Our strong findings of increased risk of severe COVID‐19 among minority racial and ethnic groups are in contrast with some earlier studies suggesting that among people testing positive for COVID‐19, risk of hospitalization and death were similar among White, Hispanic, and Black patients.^11^, ^15^ In our study, risk of hospitalization, ICU admission, and death were higher among minority racial and ethnic groups compared with White among people who tested positive. Furthermore, among hospitalized people, risk of ICU admission and death were higher among Hispanic compared with White persons.

Consistent with a recent study,^12^ we found that associations between severe COVID‐19 and comorbidities were strong for some comorbidities but were not strong for others. Based on the hazard ratios, we found strong associations of severe COVID‐19 with obesity, history of pneumonia, and diabetes and moderate associations with renal disease and essential hypertension. Severe COVID‐19 was also associated with several neurological conditions including Alzheimer's disease, demyelinating disorders, Parkinson's, and epilepsy. Associations of severe COVID‐19 with ischemic heart disease and asthma based on our measure of association were more modest.

Overall, our findings might have implications for clinical practice and for public health interventions; they identify persons at high risk of severe COVID‐19 who may most benefit from monitoring, outreach, and vaccination. Because non‐whites are at especially high risk of severe COVID‐19, even after vaccines became widely available, our race/ethnicity findings support special efforts in non‐White communities to deliver risk communication messages about COVID‐19 prevention and treatment, address vaccine hesitancy, and achieve high coverage especially in the presence of highly transmissible variants of SARS‐CoV‐2.

Our study was strengthened by the study population's size and racial, ethnic, and socioeconomic diversity. Because comorbidities were identified prior to infection with SARS‐Co‐2 using KPNC's longitudinal medical data, we have confidence that identified comorbidities preceded infection rather than being a consequence of COVID‐19.

Our study was limited by our inability to account for members' occupation, type of residence (e.g., long‐term care facility), education, income, and gender identity. We may have missed a few KPNC members with severe COVID‐19 who were hospitalized or died outside KPNC. The study was unable to assess whether risk of severe COVID‐19 outcomes varied by severity of comorbidities. KNPC membership is not representative of the US population. Although we did not stratify the analysis by vaccination status in the main analysis, we censored people at the time of vaccination in the supplemental analysis and found that risk factors did not change. Finally, we did not assess whether factors associated with severe COVID‐19 were different for vaccinated people.

In summary, our findings identify whom might benefit most from COVID‐19 prevention and treatment. Older age, non‐White race, Hispanic ethnicity, and some comorbidities—but not others—were strongly associated with severe COVID‐19.

## AUTHOR CONTRIBUTIONS

**Ousseny Zerbo:** Conceptualization; funding acquisition; investigation; methodology. **Ned Lewis:** Conceptualization; data curation; formal analysis; funding acquisition; methodology. **Bruce Fireman:** Conceptualization; funding acquisition; methodology. **Kristin Goddard:** Conceptualization; funding acquisition; project administration. **Nicola P. Klein:** Conceptualization; funding acquisition; investigation; methodology; supervision.

## CONFLICT OF INTEREST

The authors have no conflicts of interest relevant to this article to disclose. Dr. Klein has received research grants from GlaxoSmithKline, Sanofi Pasteur, Merck, Pfizer, and Protein Science (now Sanofi Pasteur). Dr. Skarbinski has received research grants from Gilead Sciences, Genentech/Roche, and Karyopharm Therapeutics. The findings and conclusions in this article are those of the authors and do not necessarily represent the official position of the US Centers for Disease Control and Prevention.

### PEER REVIEW

The peer review history for this article is available at https://publons.com/publon/10.1111/irv.12901.

## Supporting information


**Figure S1:** Weekly incidence rates of testing positive, hospitalization, admission to an intensive care unit and death. Kaiser Permanente Northern California January 1, 2020 through July 232 021

**Table S1:** Factors Associated with Hospitalization Admission to an Intensive Care Unit and Death Over time. Kaiser Permanente Northern California, January 1, 2020 ‐ July 23, 2021 (N ~ 4.6 million)
Supplemental Table 1 continued: Factors Associated with Hospitalization Admission to an Intensive Care Unit and Death Over time. Kaiser Permanente Northern California, January 1, 2020 ‐ July 23, 2021 (N ~ 4.6 million)
**Table S2:** Factors Associated with Hospitalization, Admission to an Intensive Care Unit and Death Among COVID‐19 Positive Cases (N = 219 001) with Adjustment for Age and Comorbidities ‐ Kaiser Permanente Northern California, January 1, 2020 ‐ July 23, 2021
**Table S3:** Factors Associated with Admission to an Intensive Care Unit and Death Among Hospitalized Cases (N = 16 182) With Adjustment for Age and Comorbidities‐ Kaiser Permanente Northern California, January 1, 2020‐ July 23, 2021

## Data Availability

Research data are not shared because of privacy concerns.
