# Supplementary material for: Population‐based assessment of risks for severe COVID‐19 disease outcomes
Source: Influenza Other Respir Viruses. 2021 Aug 25;16(1):159–65. doi: 10.1111/irv.12901 (PMC8652998; doi:10.1111/irv.12901)
Supplement: Supplementary file 1 — Figure S1: Weekly incidence rates of testing positive, hospitalization, admission to an intensive care unit and death. Kaiser Permanente Northern California January 1, 2020 through July 232 021 [file IRV-16-159-s002.docx]

| 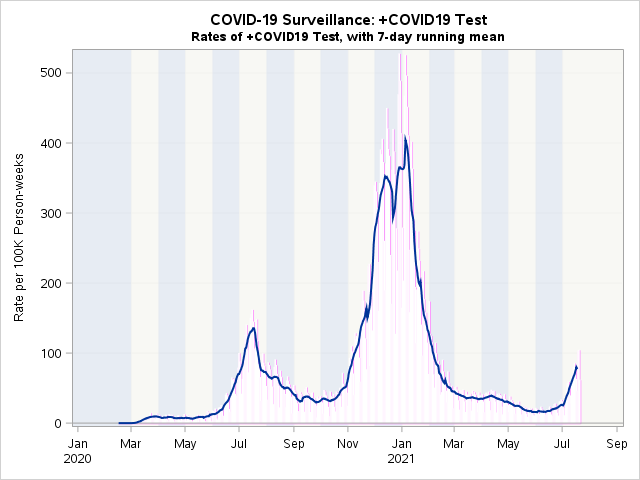  **A** | 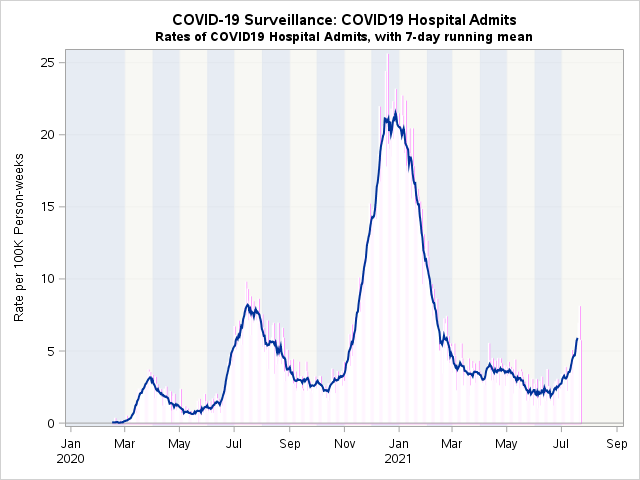  **B** |
| --- | --- |
| 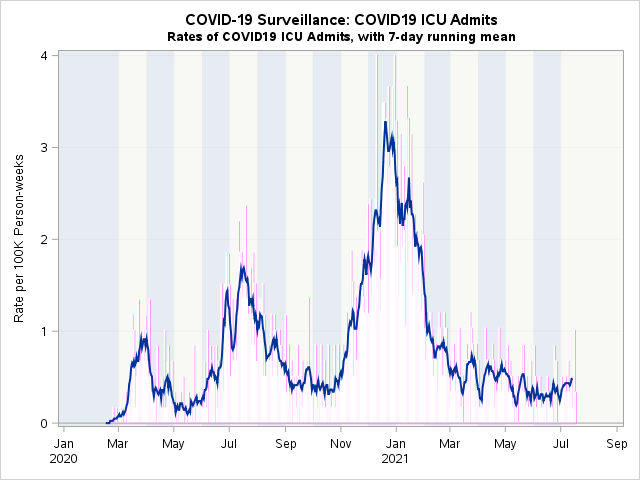  **C** | 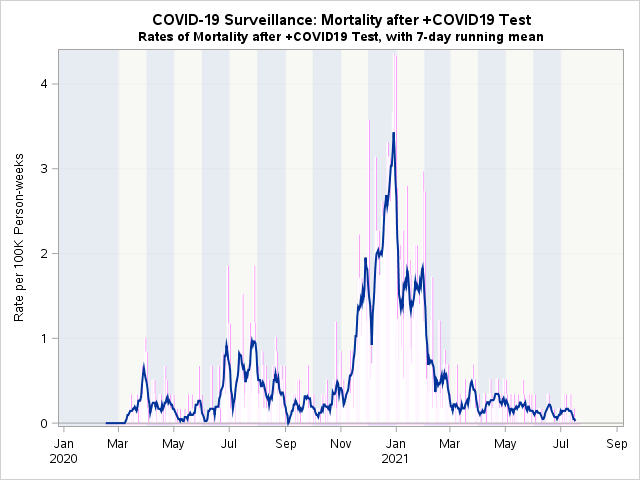  **D** |

Supplemental Figure: Weekly incidence rates of testing positive, hospitalization, admission to an intensive care unit and death. Kaiser Permanente Northern California January 1, 2020 through July 23,2021
