# Supplementary material for: Population‐based assessment of risks for severe COVID‐19 disease outcomes
Source: Influenza Other Respir Viruses. 2021 Aug 25;16(1):159–65. doi: 10.1111/irv.12901 (PMC8652998; doi:10.1111/irv.12901)
Supplement: Supplementary file 2 — Table S1: Factors Associated with Hospitalization Admission to an Intensive Care Unit and Death Over time. Kaiser Permanente Northern California, January 1, 2020 ‐ July 23, 2021 (N ~ 4.6 million) Supplemental Table 1 continued: Factors Associated with Hospitalization Admission to an Intensive Care Unit and Death Over time. Kaiser Permanente Northern California, January 1, 2020 ‐ July 23, 2021 (N ~ 4.6 million) Table S2: Factors Associated with Hospitalization, Admission to an Intensive Care Unit and Death Among COVID‐19 Positive Cases (N = 219 001) with Adjustment for Age and Comorbidities ‐ Kaiser Permanente Northern California, January 1, 2020 ‐ July 23, 2021 Table S3: Factors Associated with Admission to an Intensive Care Unit and Death Among Hospitalized Cases (N = 16 182) With Adjustment for Age and Comorbidities‐ Kaiser Permanente Northern California, January 1, 2020‐ July 23, 2021 [file IRV-16-159-s001.docx]

Supplemental Table 1: Factors Associated with Hospitalization Admission to an Intensive Care Unit and Death Over time. Kaiser Permanente Northern California, January 1, 2020 - July 23, 2021 (N ~ 4.6 million)

|  | ***Hospitalization: Hazard Ratio (95% CI)*** | | | ***Admitted to ICU^1^: Hazard Ratio (95% CI)*** | | |
| --- | --- | --- | --- | --- | --- | --- |
|  | January 1 through October 31,2020 | November 1, 2020 through February 28, 2021 | March 2021 through July 25,2021 | January 1, through October 31,2020 | November 1, 2020 through February 28, 2021 | March 2021 through July 23,2021 |
| **Sex** |  |  |  |  |  |  |
| Female | reference | reference | reference | reference | reference | reference |
| Male | **1.32 (1.24 - 1.40)** | **1.23 (1.18 -1.20)** | 0.97 (0.89 - 1.06) | **1.93 (1.68 - 0.23)** | **1.73 (1.54 - 1.96)** | **1.49 (1.16 -1.90)** |
| **Age category in year** |  |  |  |  |  |  |
| 0 – 9 | **0.05 (0.03 - 0.08)** | **0.04 (0.03 - 0.05)** | **0.05 (0.04 - 0.08)** | **0.07 (0.03 - 0.16)** | **0.27 (0.16 - 0.44)** | **0.07 (0.03 - 0.17)** |
| 10 – 19 | **0.08 (0.06 - 0.10)** | **0.06 (0.05 - 0.08)** | **0.14 (0.11 - 0.19)** | **0.09 (0.05 - 0.19)** | **0.17 (0.10 - 0.29)** | **0.13 (0.06 - 0.30)** |
| 20 – 29 | **0.45 (0.39 - 0.52)** | **0.27 (0.24 - 0.31)** | **0.60 (0.51 - 0.71)** | **0.32 (0.21 - 0.47)** | **0.31 (0.20 - 0.48)** | **0.14 (0.06 - 0.32)** |
| 30 – 39 | **0.67 (0.59 - 0.75)** | **0.39 (0.35 - 0.43)** | **0.76 (0.66 - 0.88)** | **0.49 (0.36 - 0.67)** | **0.56 (0.41 - 0.77)** | **0.36 (0.19 - 0.70)** |
| 40 – 49 | reference | **0.51 (0.47 - 0.57)** | reference | reference | reference | 0.56 (0.31 - 1.03) |
| 50 – 59 | **1.28 (1.16 - 1.41)** | **0.73 (0.67 - 0.80)** | **1.27 (1.11 - 1.45)** | **1.49 (1.20 - 1.86)** | **1.82 (1.45 - 2.28)** | 0.73 (0.41 - 1.29) |
| 60 – 64 | **1.36 (1.21 - 1.53)** | **0.87 (0.79 - 0.95)** | **1.25 (1.04 -1.49)** | **1.66 (1.28 - 2.15)** | **2.23 (1.74 - 2.86)** | 0.89 (0.48 - 1.64) |
| 65 - 69 | **1.17 (1.03 - 1.34)** | 0.91 (0.83 - 1.00) | **1.26 (1.02 - 1.55)** | **1.52 (1.15 - 2.02)** | **2.67 (2.07 - 3.44)** | 0.92 (0.48 - 1.79) |
| 70-74 | **1.26 (1.09 - 1.45)** | **reference** | **1.51 (1.21 - 1.89)** | **1.51 (1.11 - 2.05)** | **2.65 (2.03 - 3.46)** | **reference** |
| 75-79 | **1.60 (1.38 - 1.85)** | **1.18 (1.07 - 1.31)** | **1.80 (1.38 - 2.34)** | **1.96 (1.42 - 2.72)** | **3.16 (2.38 - 4.19)** | 1.34 (0.63 - 2.84) |
| 80-84 | **1.50 (1.26 - 1.79)** | **1.40 (1.25 - 1.56)** | **2.42 (1.82 - 3.21)** | **1.82 (1.24 - 2.67)** | **2.63 (1.89 - 3.67)** | **2.83 (1.40 - 5.71)** |
| 85-89 | **2.03 (1.68 - 2.46)** | **1.93 (1.72 - 2.17)** | **2.05 (1.41 - 3.00)** | 1.46 (0.87 - 2.44) | **2.40 (1.59 - 3.62)** | 1.61 (0.58 - 4.43) |
| 90+ | **2.75 (2.22 - 3.41)** | **2.20 (1.91 - 2.54)** | **2.34 (1.47 - 3.72)** | **2.33 (1.31 - 4.14)** | **2.64 (1.56 - 4.47)** | 1.31 (0.30 - 5.78) |
| **Race/Ethnicity** |  |  |  |  |  |  |
| White | reference | reference | reference | reference | reference | reference |
| Black | **2.13 (1.90 - 2.39)** | **1.61 (1.48 - 1.75)** | **2.12 (1.86 - 2.43)** | **2.70 (2.06 - 3.56)** | **1.93 (1.54 - 2.42)** | **2.23 (1.53 - 3.26)** |
| Asian | **1.76 (1.59- 1.95)** | **1.66 (1.55 - 1.77)** | **1.28 (1.10 - 1.48)** | **2.81 (2.22 - 3.55)** | **2.43 (2.02 - 2.92)** | **1.73 (1.16 - 2.56)** |
| Hawaiian/Pacific Islander | **3.73 (2.96 – 4.70)** | **2.91 (2.45 - 3.45)** | **2.04 (1.41 - 2.97)** | **4.12 (2.33 - 7.28)** | **3.60 (2.28 - 5.69)** | **4.53 (2.17 - 9.43)** |
| Native American or Alaska Native | 0.78 (0.41 - 1.51) | **1.61 (1.20 - 2.17)** | 1.32 (0.76 - 2.28) | NE | 1.29 (0.48 - 3.460) | NE |
| Multiracial | **1.65 (1.26 - 2.16)** | **1.19 (0.98 - 1.45)** | **1.58 (1.13 - 2.21)** | 1.49 (0.70 - 3.19) | **1.71 (1.03 - 2.83)** | 1.11 (0.35 - 3.52) |
| Hispanic | **3.89 (3.60 - 4.21)** | **2.57 (2.43 - 2.71)** | **1.69 (1.51 - 1.89)** | **5.85 (4.84 - 7.07)** | **3.51 (3.01 - 4.08)** | **1.73 (1.26 - 2.39)** |
| Unknown | **0.73 (0.57 - 0.92)** | **0.42 (0.34 - 0.51)** | **0.32 (0.23 - 0.45)** | 1.41 (0.86 - 2.29) | **0.45 (0.25 - 0.83)** | **0.33 (0.12 - 0.90)** |
| **Comorbidities^2^** |  |  |  |  |  |  |
| Body Mass Index^3^ |  |  |  |  |  |  |
| Underweight | **1.54 (1.15 - 2.08)** | **1.51 (1.23 - 1.85)** | 0.83 (0.45 - 1.53) | 1.63 (0.71 - 3.76) | 1.85 (0.99 - 3.44) | NE |
| Normal | reference | reference | reference | reference | reference | reference |
| Overweight | **1.29 (1.15 - 1.43)** | **1.32 (1.23 - 1.42)** | **1.50 (1.26 - 1.78)** | **1.64 (1.24 - 2.16)** | **1.50 (1.19 - 1.88)** | 1.45 (0.87 - 2.39) |
| Obese | **2.27 (2.05 - 2.52)** | **2.21 (2.06 - 2.38)** | **2.83 (2.41 - 3.32)** | **3.21 (2.46 - 4.18)** | **2.95 (2.37 - 3.67)** | **3.30 (2.08 - 5.23)** |
| Unknown | 1.08 (0.96 - 1.23) | **1.31 (1.20 - 1.43)** | **1.43 (1.20 - 1.71)** | **1.54 (1.13 - 2.11)** | **2.16 (1.66 - 2.793)** | **1.86 (1.12 - 3.08)** |
| Diabetes | **1.84 (1.71 - 1.98)** | **1.73 (1.64 - 1.82)** | **1.82 (1.61 - 2.05)** | **2.10 (1.79 - 2.47)** | **1.96 (1.70 - 2.25)** | **1.95 (1.43 - 2.67)** |
| Essential hypertension | **1.16 (1.07 - 1.25)** | **1.29 (1.22 - 1.37)** | **1.18 (1.05 - 1.32)** | **1.24 (1.04 - 1.47)** | **1.54 (1.32 - 1.80)** | **1.56 (1.13 - 2.15)** |
| Renal disease | **1.36 (1.24 - 1.49)** | **1.45 (1.37 - 1.54)** | **1.28 (1.09 - 1.50)** | **1.53 (1.26 - 1.85)** | **1.80 (1.54 - 2.11)** | 1.02 (0.68 - 1.53) |
| Asthma | **1.16 (1.07 - 1.25)** | **1.15 (1.09 - 1.22)** | 1.08 (0.96 - 1.22) | 1.10 (0.92 - 1.32) | **1.23 (1.06 - 1.43)** | 0.98 (0.69 - 1.38) |
| Ischemic heart disease | **1.11 (1.00 - 1.24)** | **1.16 (1.08 - 1.25)** | **1.42 (1.18 - 1.71)** | 1.07 (0.85 - 1.36) | 1.09 (0.91 - 1.31) | 1.17 (0.73 - 1.87) |
| COPD^4^ | **1.24 (1.08 - 1.42)** | **1.38 (1.26 - 1.50)** | **1.62 (1.29 - 2.02)** | 1.17 (0.85 - 1.61) | 1.05 (0.81 - 1.35) | **2.36 (1.42 - 3.91)** |
| Pneumonia (history) | **1.72 (1.55 - 1.91)** | **1.71 (1.60 - 1.84)** | **1.29 (1.07 - 1.55)** | **1.77 (1.41 - 2.22)** | **2.06 (1.72 - 2.46)** | **1.32 (0.82 - 2.10)** |
| Cerebral infarction | **1.31 (1.08 - 1.58)** | **1.48 (1.31 - 1.67)** | **1.55 (1.14 - 2.13)** | 0.93 (0.58 - 1.51) | **1.84 (1.38 - 2.46)** | **3.01 (1.67 - 5.45)** |
| Alzheimer disease | **3.91 (3.24 - 4.72)** | **3.21 (2.82 - 3.66)** | **1.78 (1.08 - 2.92)** | **2.28 (1.28 - 4.05)** | **1.78 (1.07 - 2.97)** | **NE** |
| Parkinson disease | **1.98 (1.47 - 2.65)** | **1.64 (1.33 - 2.01)** | **2.39 (1.40 - 4.09)** | 1.31 (0.58 - 2.95) | 1.56 (0.88 - 2.78) | 1.95 (0.47 - 8.00) |
| Extrapyramidal and movement disorders | **1.09 (0.90 - 1.33)** | **1.30 (1.15 - 1.47)** | 1.09 (0.79 - 1.52) | 1.06 (0.66 - 1.68) | 1.27 (0.91 - 1.77) | 1.01 (0.41 - 2.49) |
| Demyelinating disorders | **2.68 (1.83 - 3.91)** | 1.37 (0.94 - 1.98) | 1.36 (0.65 - 2.86) | 1.68 (0.54 - 5.24) | **2.31 (1.03 - 5.17)** | **NE** |
| Epilepsy | **1.60 (1.29 - 1.98)** | **1.88 (1.63 - 2.16)** | 0.95 (0.63 - 1.44) | **2.27 (1.48 - 3.49)** | **1.74 (1.18 - 2.56)** | 0.81 (0.26 - 2.56) |
| **Type of insurance** |  |  |  |  |  |  |
| Subsidized insurance^5^ | **1.55 (1.41 - 1.71)** | **1.48 (1.38 - 1.59)** | **1.29 (1.13 - 1.46)** | **1.79 (1.45 - 2.21)** | **1.64 (1.35 - 1.98)** | **1.89 (1.35 - 2.63)** |

^1^ICU = intensive care unit. ^2^Based on ICD 10 codes recorded in medical record ^3^BMI categories: Underweight BMI = <18.5, Normal weight = BMI between 18.5–24.9, Overweight = BMI between 25–29.9, Obesity = BMI ≥of 30. ^4^COPD = chronic obstructive pulmonary disease. ^5^Includes those who cannot afford the regular insurance rates and are not covered by commercial insurance or Medicare.

NE= not estimated

Supplemental Table 1 continued: Factors Associated with Hospitalization Admission to an Intensive Care Unit and Death Over time. Kaiser Permanente Northern California, January 1, 2020 - July 23, 2021 (N ~ 4.6 million)

|  | ***Mortality: Hazard Ratio (95% CI)*** | | |
| --- | --- | --- | --- |
|  | January 1, through October 31,2020 | November 1, 2020 through February 28, 2021 | March 2021 through July 23,2021 |
| **Sex** |  |  |  |
| Female | reference | reference | reference |
| Male | **1.71 (1.40 - 2.09)** | **1.81 (1.58 - 2.07)** | **1.53 (1.03 - 2.28)** |
| **Age category in year** |  |  |  |
| 0 – 9 | NE | NE | NE |
| 10 – 19 | **0.08 (0.01 - 0.61)** | NE | NE |
| 20 – 29 | **0.23 (0.07 - 0.79)** | **0.03 (0.00 - 0.24)** | NE |
| 30 – 39 | 0.40 (0.16 - 1.02) | **0.22 (0.10 - 0.474)** | **0.23 (0.05 - 1.09)** |
| 40 – 49 | reference | reference | reference |
| 50 – 59 | **3.41 (1.96 - 5.92)** | **2.73 (1.89 - 3.94)** | **2.31 (1.00 - 5.34)** |
| 60 – 64 | **5.09 (2.87 - 9.05)** | **4.12 (2.81 - 6.06)** | **4.02 (1.63 - 9.90)** |
| 65 - 69 | **4.82 (2.66 - 8.73)** | **5.68 (3.88 - 8.30)** | **4.87 (1.85 - 12.77)** |
| 70-74 | **6.17 (3.41 - 11.16)** | **7.24 (4.95 - 10.59)** | **6.34 (2.38 - 16.95)** |
| 75-79 | **8.53 (4.69 - 15.50)** | **10.24 (6.97 - 15.05)** | **13.88 (5.33 - 36.12)** |
| 80-84 | **9.11 (4.89 - 16.99)** | **11.77 (7.89 - 17.55)** | **24.39 (9.38 - 63.39)** |
| 85-89 | **16.40 (8.81 - 30.52)** | **19.55 (13.03 - 29.33)** | **19.88 (6.52 - 60.63)** |
| 90+ | **36.06 (19.44 - 66.88)** | **34.22 (22.52 - 51.99)** | **47.58(16.13 - 140.32)** |
| **Race/Ethnicity** |  |  |  |
| White | reference | reference | reference |
| Black | **2.43 (1.76 - 3.36)** | **1.40 (1.08 - 1.83)** | 1.73 (0.93 - 3.22) |
| Asian | 1.01 (0.70 - 1.44) | **1.66 (1.35 - 2.03)** | 1.22 (0.62 - 2.40) |
| Hawaiian/Pacific Islander | 1.45 (0.46 - 4.58) | 1.39 (0.65 - 2.94) | 2.89 (0.69 - 12.14) |
| Native American or Alaska Native | 1.82 (0.45 - 7.36) | 1.81 (0.75 - 4.37) | **NE** |
| Multiracial | 1.70 (0.83 - 3.48) | **1.69 (1.06 - 2.69)** | 2.10 (0.65 - 6.82) |
| Hispanic | **3.18 (2.51 - 4.03)** | **2.58 (2.19 - 3.03)** | **1.68 (1.02 - 2.77)** |
| Unknown | 0.46 (0.14 - 1.45) | **0.30 (0.11 - 0.80)** | 0.31 (0.04 - 2.29) |
| **Comorbidities^2^** |  |  |  |
| Body Mass Index^3^ |  |  |  |
| Underweight | 1.45 (0.75 - 2.79) | 1.31 (0.76 - 2.28) | 0.93 (0.12 - 7.24) |
| Normal | reference | reference | reference |
| Overweight | 0.80 (0.59 - 1.07) | 1.14 (0.93 - 1.40) | 1.36 (0.68 - 2.75) |
| Obese | **1.39 (1.05 - 1.850)** | **2.11 (1.72 - 2.58)** | **2.62 (1.34 - 5.13)** |
| Unknown | 1.17 (0.78 - 1.74) | 1.23 (0.90 - 1.67) | 1.92 (0.87 - 4.25) |
| Diabetes | **1.64 (1.32 - 2.04)** | **1.58 (1.37 - 1.834)** | **2.31 (1.47 - 3.61)** |
| Essential hypertension | **1.62 (1.24 - 2.13)** | **1.42 (1.19 - 1.70)** | 1.19 (0.71 – 2.00) |
| Renal disease | **1.56 (1.23 - 1.98)** | **1.65 (1.41 - 1.93)** | 1.21 (0.74 - 1.98) |
| Asthma | 1.06 (0.82 - 1.38) | 1.12 (0.94 - 1.32) | 1.01 (0.59 - 1.74) |
| Ischemic heart disease | **1.32 (1.03 - 1.70)** | 1.14 (0.96 - 1.35) | **1.80 (1.08 - 2.982)** |
| COPD^4^ | 1.14 (0.82 - 1.59) | 1.22 (0.98 - 1.51) | **1.92 (1.04 - 3.54)** |
| Pneumonia (history) | **1.97 (1.52 - 2.55)** | **1.88 (1.58 - 2.25)** | 1.03 (0.54 - 1.97) |
| Cerebral infarction | 1.21 (0.77 - 1.88) | **1.63 (1.23 - 2.15)** | **2.24 (1.05 - 4.76)** |
| Alzheimer disease | **4.66 (3.31 - 6.57)** | **3.91 (3.01 - 5.083)** | 1.91 (0.67 - 5.40) |
| Parkinson disease | **2.71 (1.55 - 4.77)** | 1.55 (0.97 - 2.49) | 1.96 (0.47 - 8.14) |
| Extrapyramidal and movement disorders | 0.59 (0.31 - 1.11) | **1.47 (1.09 - 1.97)** | 0.86 (0.27 - 2.76) |
| Demyelinating disorders | **4.18 (1.56 - 11.24)** | 1.49 (0.48 - 4.62) | NE |
| Epilepsy | **2.28 (1.39 - 3.74)** | **1.67 (1.13 - 2.49)** | 0.51 (0.07 - 3.73) |
| **Type of insurance** |  |  |  |
| Subsidized insurance^5^ | 1.00 (0.65 - 1.54) | **1.71 (1.35 - 2.17)** | 1.33 (0.67 - 2.61) |

^1^ICU = intensive care unit. ^2^Based on ICD 10 codes recorded in medical record. ^3^BMI categories: Underweight BMI = <18.5, Normal weight = BMI between 18.5–24.9, Overweight = BMI between 25–29.9, Obesity = BMI ≥30. ^4^COPD = chronic obstructive pulmonary disease. ^5^Includes those who cannot afford the regular insurance rates and are not covered by commercial insurance or Medicare.

NE= not estimated

Supplemental Table 2: Factors Associated with Hospitalization, Admission to an Intensive Care Unit and Death Among COVID-19 Positive Cases (N = 219001) with Adjustment for Age and Comorbidities - Kaiser Permanente Northern California, January 1, 2020 - July 23, 2021

|  | ***Hospitalization*** | ***Admitted to ICU*** | ***Deaths*** |
| --- | --- | --- | --- |
|  | Odds Ratio (95% CI) | Odds Ratio (95% CI) | Odds Ratio  (95% CI) |
| **Sex** |  |  |  |
| Female | reference | reference | reference |
| Male | **1.31 (1.26 - 1.36)** | **1.77 (1.61 - 1.93)** | **1.83 (1.64 – 2.04)** |
| **Race/Ethnicity** |  |  |  |
| White | reference | reference | reference |
| Black | **1.53 (1.43 - 1.64)** | **1.64 (1.39 – 1.94)** | **1.39 (1.14 - 1.68)** |
| Asian | **1.61 (1.52 - 1.71)** | **2.25 (1.96 - 2.58)** | **1.45 (1.22 - 1.72)** |
| Hawaiian/Pacific Islander | **1.76 (1.52 - 2.05)** | **2.17 (1.56 - 3.00)** | 0.87 (0.49 - 1.55) |
| Native American or Alaska Native | 1.08 (0.83 - 1.41) | 0.75 (0.33 - 1.70) | 1.15 (0.53 - 2.49) |
| Multiracial | **1.27 (1.07 - 1.50)** | **1.79 (1.24 - 2.59)** | **1.60 (1.08 - 2.37)** |
| Hispanic | **1.30 (1.24 - 1.36)** | **1.72 (1.54 – 1.93)** | **1.42 (1.24 - 1.62)** |
| Unknown | **0.42 (0.36 - 0.48)** | **0.59 (0.41 - 0.85)** | **0.49 (0.27 - 0.87)** |
| **Type of insurance** |  |  |  |
| Subsidized insurance | **1.28 (1.20 - 1.36)** | **1.37 (1.19 - 1.58)** | 1.21 (0.99 - 1.49) |

Supplemental Table 3: Factors Associated with Admission to an Intensive Care Unit and Death Among Hospitalized Cases (N = 16182) With Adjustment for Age and Comorbidities- Kaiser Permanente Northern California, January 1, 2020- July 23, 2021

|  | ***Admitted to ICU*** | ***Deaths*** |
| --- | --- | --- |
|  | Odds Ratio (95% CI) | Odds Ratio  (95% CI) |
| **Sex** |  |  |
| Female | reference | reference |
| Male | **1.44 (1.31 - 1.59)** | **1.56 (1.38 - 1.76)** |
| **Race/Ethnicity** |  |  |
| White | reference | reference |
| Black | **1.20 (1.01 - 1.44)** | 1.05 (0.85 - 1.31) |
| Asian | **1.66 (1.43 - 1.93)** | 1.22 (1.01 - 1.47) |
| Hawaiian/Pacific Islander | **1.44 (1.02 - 2.05)** | 0.73 (0.41 - 1.32) |
| Native American or Alaska Native | 0.64 (0.27 - 1.51) | 0.86 (0.33 - 2.20) |
| Multiracial | **1.60 (1.08 - 2.38)** | 1.16 (0.73 - 1.84) |
| Hispanic | **1.47 (1.30 - 1.67)** | **1.37 (1.18 - 1.58)** |
| Unknown | 1.30 (0.88 - 1.93) | 0.91 (0.45 - 1.84) |
| **Type of insurance** |  |  |
| Subsidized insurance | 1.18 (1.01 - 1.37) | 1.09 (0.87 - 1.35) |
